# Nurses' Workplace Social Capital and the Influence of Transformational Leadership: A Theoretical Perspective

**DOI:** 10.3389/fpubh.2022.855278

**Published:** 2022-06-13

**Authors:** Jia-Min Xu, Azadeh T. Stark, Bi-He Ying, Zheng-Mei Lian, Yang-Sheng Huang, Rui-Ming Chen

**Affiliations:** ^1^Department of Nursing Sciences, School of Medicine, Lishui University, Lishui, China; ^2^Department of Pathology and Laboratory Medicine, Henry Ford Health System, Detroit, MI, United States; ^3^School of Interdisciplinary Studies, University of Texas at Dallas, Richardson, TX, United States

**Keywords:** social capital, nurses, workplace, transformational leadership, theoretical analysis

## Abstract

Workplace social capital is the relational network, created by respectful interactions among members of a workforce, can contribute to the formation of a wholesome psychological work environment in an organization. Nurses' workplace social capital is a derivative of the workplace social capital, formed because of the complex interactions among the nursing and between the other healthcare professionals. Transformational leadership is a style of leadership that addresses the emotional wellbeing of its workforce and inspires shared group ethics, norms, and goals. The philosophy of transformational leadership is grounded on the premise of workforce as human beings with specific needs. Transformational leadership has been confirmed as a strong predictor of nurses' workplace social capital. Meanwhile, it is of an academic and/or healthcare industry operational value to scholarly assess and discern the theoretical influence of transformational leadership on nurses' workplace social capital. In this paper, we have attempted to explore the associations between transformational leadership and nurses' workplace social capital from a theoretical perspective. We have discussed the importance of each sub-dimension of transformational leadership (modeling the way, inspiring a shared vision, challenging the process, enabling others to act and encouraging the heart) in building up the social capital relational network. Finally, we have proposed a graphic framework of our analysis to facilitate understanding of the associations between the transformational leadership and nurses' workplace social capital, in formation of a healthy work environment which is the foundation for efficiency and productivity of the workforce.

## Introduction

Nursing professionals account as the largest segment of the healthcare workforce. Public ranks nursing as the most trusted profession (1). The term “wingless angels” has been used to describe the importance of these professionals in the delivery of healthcare services. Yet, these “wingless angels” are humans and, therefore, susceptible to negative aspects, i.e., stress, psychological duress, mental fatigue, and exhaustion, and mis-communication, in a work-environment; these negative aspects can influence the nursing professionals' efficiency and effectiveness in the delivery of healthcare services (2).

Workplace social capital is an intangible but a formidable source that can improve effectivity and productivity of a workforce (3). Nurses' workplace social capital, a derivative of the concept of workplace social capital, has promising impacts beyond nursing professionals (4, 5). Value of nurses' social capital in efficiency and effectiveness of the nursing workforce was well-demonstrated during the peak of the COVID-19 epidemic; workplace social capital mitigated psychological stress and fortified professional identity of the nursing professionals, particularly those at the frontline (6). The importance of strengthening workplace social capital for nursing professionals should be reviewed and reverberated.

Transformational leadership can strongly influence the development of nurses' workplace social capital (7). This style of leadership orients itself on relationship building and has become one of the predominant styles of leadership in the 21st century (8–10). Transformative leaders concern themselves with physical and psychological wellbeing of their workforce and are dedicated to creating productive work environments, intertwined with harmonious social capital relational networks. Findings from our previous study of the association between nurse managers' transformational leadership and nurses' social capital yielded that transformational leadership abilities of nurse managers was a strong predictor of nurses' workplace social capital (7). However, there is a lack of perspective analysis of this relationship in academia. Our paper, therefore, is to address this academic deficiency.

The concept of social capital first was used by Hanifan in 1916 to depict the relationships and interactions within a community (11). The seminal work of Pierre Bourdieu, published in 1986 under the title of “The Forms of Capital,” reinvigorated the scientific interest in the concept of social capital and instigate the contemporary research on this topic (12–17).

The pivotal role of social capital at workplace (workplace social capital) has been pronounced and palpated across the globe due to intra and inter connectivity within and among the workforce and the increase in demand for productivity (18). The collection of social capital in the workplace functions as a powerful magnet in attracting employees netted in the relational network and nourishes their interactions, the key factor for the formation of healthy and productive workforce.

The concept of “social capital” was introduced into the field of academic nursing in the mid-1990s (19). In 2002 Pesut in response to the tragedies of 9/11 in New York City, called for operation of workplace social capital among the nursing professionals (20). E.A. Read, in 2014, first coined the concept of “nurses' workplace social capital” in her pioneer concept analysis work (21). Studies have shown positive consequences of workplace social capital; the spectrum of positive gains varies from improving health status of the nursing professionals to the quality of care of patients, and safety in operation at every aspect of the delivery of healthcare services (4, 5).

Findings from our concept analysis yielded the definition of nurses' workplace social capital as the “*relational network configured by respectful interactions among nursing professionals and between the other healthcare professionals. These interactions are characterized by the norms of trust, reciprocity, shared understanding, and social cohesion*” (22). This definition highlights the five core attributes of nurses' workplace social capital: (1) Relational Network; (2) Trust; (3) Reciprocity, (4) Shared Understanding and (5) Social Cohesion; meanwhile, nurses' workplace social capital has been categorized into three pillars: (1) Type (bonding, bridging, and linking); (2) Component (structural and cognitive) and (3) Level (group and individual) (22). Bonding and bridging indicate relationships at a horizontal direction, e.g., the relationships among nursing professionals and between the other healthcare professionals in the workplace; while, liking represents the vertical relationships, e.g., interactions between nurses and nurse managers. Structural social capital defines the configuration of the relational network and cognitive social capital describes the assets that are embedded in the relational network. Finally, nurses' workplace social capital can be stratified into individual and group levels. Of all the three pillars of the nurses' workplace social capital, Type and Component of workplace social capital most have been topics of academic research and discussions in the nursing field.

The pillar of Type (linking, bonding, and bridging) suggests the direction of the relationships in the workplace social capital network. The current presentation of nurses' social capital displays Type as a standalone pillar. We would like to propose to reposition of placing the three segments (linking, bonding, and bridging) of Type under the pillar of Component, within the classification of structural social capital. We rest our proposition based on the rational that these three segments (linking, bonding, and bridging) describe the structural directions of workplace social capital relational network. We have allocated the rest of this manuscript to logically assert our proposal.

## Transformational Leadership: A Collection of Relational-Oriented Leadership

The importance of leadership in human interactions and relationships cannot be denied. Since the dawn of civilization various models and philosophies of leadership have been proposed, implemented, and practiced. The most current model, transformational leadership, introduced in the late 1970 s, has been put into practice by many organizations and has become one of the predominant relationally focused leadership styles (8–10). Transformative leaders admire and highlight harmonious relationships with their constituencies and believe that they have specific social needs and strong desires for belonging. J. M. Burns is credited as the first to formally discuss transformational leadership in his 1978 seminal publication (23). He pointed out that the action of transformative leading occurs when leaders and their constituencies pursue a higher level of motivation and morality and attempt to share group goals. Transformational leaders are considered visionary individuals with creative insights on leading personnel, management of challenges and the organization (9, 24).

Kouzes and Posner used the term “exemplary leader” as the synonym of transformational leaders and proposed the Five Practice Model which was derived from analysis and integration of a vast sets of quantitative and qualitative data (25). Kouzes and Posner described transformational leadership as a collection of exemplary practices and behaviors that can be summarized into Five Practice Model: (*1) Modeling the Way; (2) Inspiring a Shared Vision; (3) Challenging the Process; (4) Enabling Others to Act and (5) Encouraging the Heart* (25).

### Modeling the Way

This component is the principal behavior of transformational leaders to win respect and trust of their constituencies; they strive to become an exemplar figure for others by portraying their genuine respect for every member of their organizations and developing goals and objectives that are shared by their constituencies.

### Inspiring a Shared Vision

Transformational leaders believe in and are able to effectively connect with members of their team. This ability of transformational leaders enables their teams to actively take part in configuring and developing organizational blueprint and roadmaps for future developments.

### Challenging the Process

Transformational leaders do not shun away from opportunities to address unpredictable challenges. They are creative individuals who assume responsibilities for mistakes and failures. They are able to sow seeds of knowledge from failures and mistakes.

### Enabling Others to Act

A harmonious work environment is one of the crucial ingredients for cooperation among members of a team. Leaders with transformational style are skillful in creating wholesome atmospheres which not only promote active cooperation of team members, but also empower them with freedom of self-determination.

### Encouraging the Heart

The last domain of transformation leadership is the practice of encouragement and motivation of constituencies. In the habit of developing a winning team, transformational leaders inspire their constituencies to achieve their pinnacle of professional productivity; furthermore, transformational leaders are willing to spend time to recognize and celebrate success of their constituencies and cement the sense of belonging among members of their teams.

Leadership styles of nurse managers influence their relationships with the nursing professionals (26, 27). Research supports that transformational leadership skills can be used as a predictive measure of workplace social capital (28–30). Previously we reported that nurse managers' transformational leadership is positively associated with nurses' workplace social capital (7).

## Transformational Leadership: The “Cradle” for Nurses' Workplace Social Capital

Nursing is a human-oriented profession that is influenced by the complexity and complicated human relationships (31). Transformational leadership by virtue of its relational orientation is particularly suitable for building up social capital relational network at workplace (28). Transformational leadership is the “cradle” for constructing nurses' workplace social capital relational network and nurse leaders who embrace the philosophy of transformational leadership hold the key positions in constructing workplace social capital relational network.

**Figure 1:**
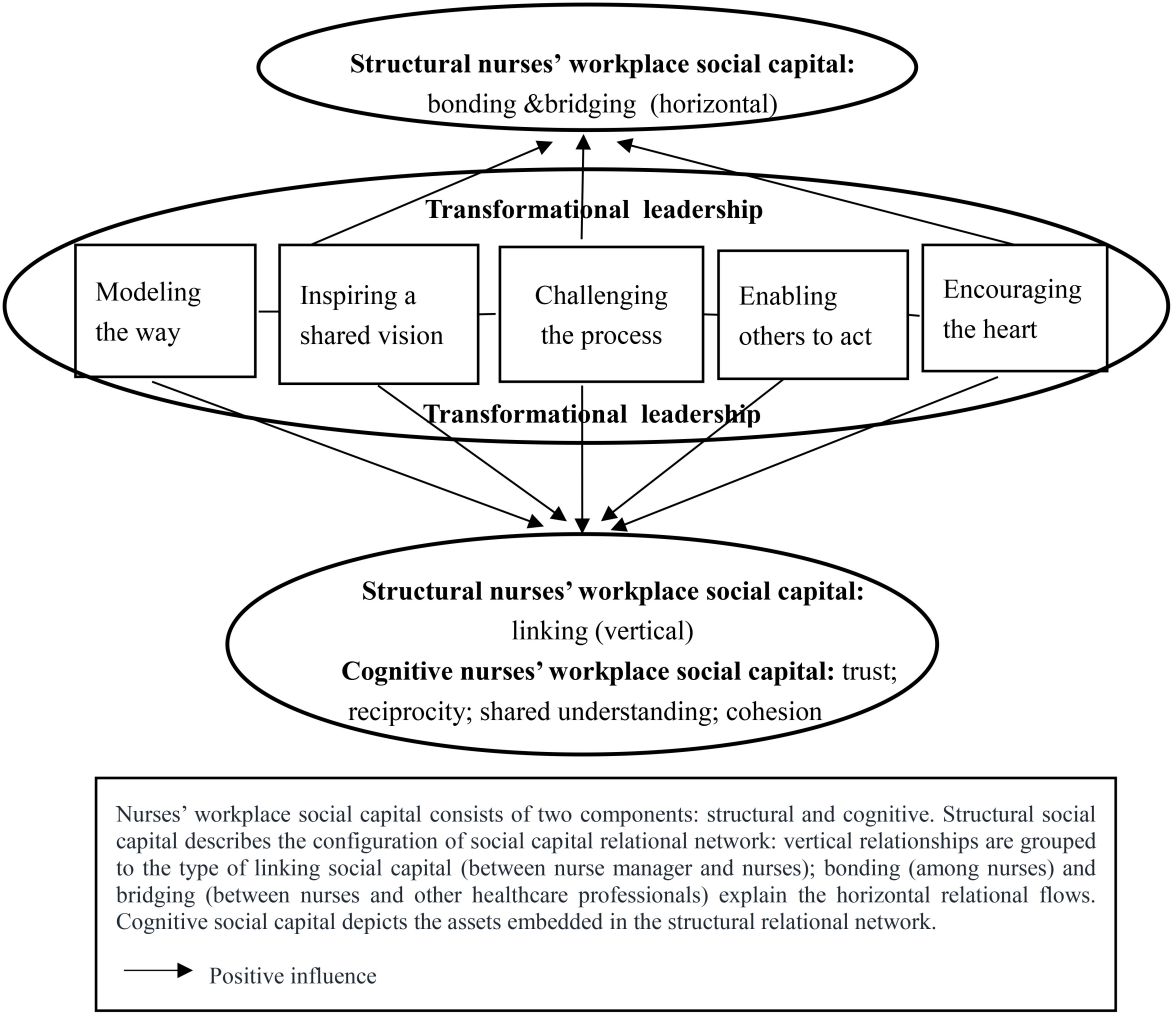
Transformational leadership and nurses' workplace social capital.

We conducted several rounds of team discussions and consultations with clinical nursing experts between May and September of 2021 in developing our theoretical explanation about the influence of the five types of transformational leadership practices on the core components and attributes of nurses' workplace social capital. A graphic framework has been developed to provide a complementary visual illustration (32) to facilitate the understanding of our theoretical propositions (Figure 1).

The practice of *Modeling the Way* is the fundamental characteristic of transformational leaders. Transformational leaders strive to win respect and gain the trust of their constituencies (25, 33). Transformational leaders are described as “visionary heroes” with creative thoughts and visionary insights about management, operation and guiding of the workforce (24, 34). The exemplar leaders influence the behaviors of their constituencies because of their credibility and their abilities in establishing trust with their workforce. *This Trust and credibility leads into the formation of shared values (shared understanding), which are the two main attributes of nurses' workplace social capital* (22).

The nurse manager is the closest leader to the other nursing professionals at any ward. If we make the analogy of clinic workplace as a ship sailing on the open sea, the nurse manager with transformational leadership style would be the sagacious navigator. She/he sets up the shared standards of excellence (shared understanding) in the ward and clarifies uncertainties so that the other nursing professionals can reach to a higher level of professional destinations. Nurse managers with transformational leadership style can be trusted and create mutual trust among nursing professionals that can lead to the affirmation of a strong vertical relational network (*structural social capital: linking*). The *cognitive social capital (e.g., trust, shared understanding)* embedded in this strong and healthy vertical social capital network will be incubated and flourished.

The second component of transformational leadership is *Inspiring a Shared Vision*. The shared vision is the blueprint for the team's future. A transformational leader can inspire a shared vision through specific practices, e.g., clear communication and enthusiasm about a hopeful future and possibilities (25); s*hared vision (understanding)* is also an important attribute of cognitive social capital. In some healthcare settings, nurse professionals are still considered as the subordinate group and are prone to workplace discrimination (35). Nursing professionals, therefore, sometimes feel hopeless about their careers which can reduce their confidence in their professional developments; absence of this confidence can be a barrier to accepting and acknowledging constructive suggestions and ideas from the other professional groups. A transformational nurse manager can act as the enabling factor in envisioning a shared promising future and encouraging the nursing professionals to turn the “unpromising” situations into opportunities for professional growth and development. In other words, a transformational nurse manager, by actions, can demonstrate the meaning of the old saying “when life gives you a lemon, make lemonade!” In return, the nursing professionals will be more prone to open their minds in adopting new insights from the other healthcare professionals in pursuing a great shared vision with others. Both vertical and horizontal interactions of a team (*structural social capital: linking, bonding, and bridging*) can be strengthened with increased enthusiasm for a shared future blueprint (28); *team cohesion* (*cognitive social capital*) is raised simultaneously along with these positive interactions.

The nursing workforce has been faced with tremendous challenges as the results of the rapid changes and responsibilities that have been introduced into the profession since the turn of the 21st century (36). Transformational leaders who *challenge the process* always seek out opportunities for changing their own status quo and that of others (33). Transformational nurse managers are not afraid of challenging or testing their own capabilities in risk-taking and actively helping the other nursing professionals to create innovative ways of thinking and acting; also, they are brave enough to face potential risks and failures at work which they cherish as precious learning opportunities. These behaviors form an active and positive atmosphere which enables the nursing professionals to bond and to reciprocatively support each other (*structural social capital: bonding; cognitive social capital: reciprocity*) in addressing and overcoming the workplace challenges and obstacles. Challenges in the delivery of healthcare services require cooperation across disciplines in finding practical and plausible answers to the problems on hand (37). This cooperation will be more effective and efficient with the establishment of *bridging social capital*. Cooperation and interactions are the fundamental resources for creating horizontal and vertical relationships (*structural social capital: linking, bonding, and bridging*) and generating valuable cognitive assets e.g., *trust, reciprocity, cohesion* (*cognitive social capital*).

The practice of E*nabling Others to Act* is another important resource for building the workplace social capital. Group accomplishment cannot be fulfilled by the leaders alone. Transformational nurse leaders always strive to build good relationships with people at workplace (38). Nursing professionals, the largest portion of the healthcare workforce, are diverse in cultural background, racial/ethnic heritage, and sexual orientation (26). Transformational nurse managers show patience and acceptance for diversity, delight in listening to and respect for opinions that are not fully in congruent with their own. They have and show sincere considerations for others as human beings with specific needs. Transformational nurse managers are able to generate and strengthen *team cohesion (cognitive social capital)* through tolerance and consideration. The transformational nurse manager seeks opportunities for staff's personal and professional growth and performance extraordinaire (33, 39). Such efforts lead to *vertical trust and mutual trust (cognitive social capital)* between the transformational nurse managers and the other nursing professionals (*structural social capital: linking*) because the authenticity of their leaders gets exuded through sincere actions.

Finally, the practice of *Encouraging the Heart* of the transformational nurse managers contributes to the formation of nurses' workplace social capital. They acknowledge and celebrate group and individual victories in inspiring the team spirit (33). They heartedly respect the nursing professionals' contributions to the goals and objectives of their teams; these heart-felt respects and recognitions are conveyed timely, not only in private but also via public celebratory events. These celebratory events, when appropriate and professionally suitable, can enhance *the sense of group belongings (cognitive social capital: cohesion) (22) and structural social capital relationships (linking and bonding*) *in the team*; furthermore, the transformational nurse manager can apply the accomplishments of the nursing professionals in developing and/or strengthening professional interactions and camaraderie between the nursing professionals and the other healthcare providers (*structural social capital: bridging*).

In summary, transformational leaders can develop and establish intimate and yet professionally appropriate emotional ties, high-level trust, team interaction and shared visions with the members of their teams (29, 40). They mobilize their constituencies by focusing on the welfare and wellbeing of each individual and the team with the objective of enabling them to know and feel of being an important part of a greater picture, their community (41). A nurse transformational manager plays a crucial and an essential role in “humanizing” a workplace; we believe this “humanization” is the “cradle” for the development of harmonious relationships in the network of nurses' workplace social capital.

## Conclusions

Nursing professionals are the backbone of the healthcare industry and a strong force to improve the cost effectiveness of the delivery of healthcare services. Nursing professionals communicate and cooperate with each other and the other healthcare professionals within the complex social capital network. A well-woven nurses' workplace social capital network is the tenet for a healthy work environment and ensures the efficiency and effectiveness of delivery of healthcare services. Measures to enhance nurses' workplace social capital should be put on the agenda by institutional administrations to assert flourishing of nurses' workplace social capital; furthermore, other workplace measures should be addressed through legislations and implementation of healthcare and public policies to ascertain preparedness of the healthcare industry for future challenges. Empirical research has shown that the relational-oriented transformational leadership provides an effective way to strengthening the workplace social capital for nursing professionals.

In our paper, we have proposed possible mechanisms of transformational leadership on nurses' workplace social capital from a theoretical perspective. The detailed analysis triangulating with the empirical results should formulate constructive suggestions for nurse administrators and policymakers when striving to develop workplace social capital. Additionally, our analysis should shed light on clues for researchers in other scientific fields when attempting to explore the interactions between leadership and social capital in their domains. The main strength of our study is its proposed theoretical perspective mechanism of influence of the transformation leadership on nurses' workplace social capital. Our perspective analysis has its limitation. Our theoretical work was designed to address the theoretical deficiency of the interaction between transformational leadership and nurses' workplace social capital. In consequence, the influence of variables such as task structure, or leader position power, or impact of situation type were not evaluated. Meanwhile, our work is a prelude for future theories for each specific nursing context, pillared by transformational leadership and nurses' workplace social capital. Future research should evaluate influence of elements such as leader and subordinates' relationship and/or task structure and their interaction with these two concepts. Despite its limitation, our work shed light on constructive suggestions for nurse administrators and policymakers when striving to develop workplace social capital for nursing professionals.

## Data Availability Statement

The original contributions presented in the study are included in the article/supplementary material, further inquiries can be directed to the corresponding author/s.

## Author Contributions

J-MX and AS conceptualized the framework of the manuscript. All authors have contributed to the development and approval of the manuscript.

## Funding

This work was supported by Public Welfare Technology Application Research Project of Lishui Science & Technology Bureau, China (Project No. 2022GYX65).

## Conflict of Interest

The authors declare that the research was conducted in the absence of any commercial or financial relationships that could be construed as a potential conflict of interest.

## Publisher's Note

All claims expressed in this article are solely those of the authors and do not necessarily represent those of their affiliated organizations, or those of the publisher, the editors and the reviewers. Any product that may be evaluated in this article, or claim that may be made by its manufacturer, is not guaranteed or endorsed by the publisher.
